# Recuperação Percutânea de Vegetação em Paciente Pediátrico com Persistência do Canal Arterial: Uma Nova Técnica para Evitar Toracotomia

**DOI:** 10.36660/abc.20240010

**Published:** 2024-10-08

**Authors:** Miguel Fabian Barrerra-Colín, José Luis Colín-Ortiz, Carlos Alfonso Corona-Villalobos

**Affiliations:** 1 Instituto Nacional de Pediatria Ciudad De Mexico México Instituto Nacional de Pediatria, Ciudad De Mexico – México

**Keywords:** Endocardite, Intervenção Coronária Percutânea, Permeabilidade do Canal Arterial, Criança

## Abstract

A endocardite infecciosa na população pediátrica é uma condição rara que pode ou não estar associada a uma cardiopatia congênita. As modalidades de tratamento atuais baseiam-se na antibioticoterapia de longo prazo e na ressecção cirúrgica como primeira opção para casos de vegetação persistente. Apresentamos um caso de recuperação percutânea bem-sucedida de vegetação em canal arterial patente, que se estendia ao tronco da artéria pulmonar em um paciente pediátrico. Este é o primeiro relato na literatura desse tipo de extração de vegetação no canal arterial sem toracotomia.

## Introdução

Massas intra ou extracardíacas podem ser tumores, trombos ou vegetações.^1^ As vegetações podem ocorrer em pacientes sem doença cardíaca subjacente, mas na maioria dos casos estão associadas a doenças cardíacas congênitas ou adquiridas. A incidência de endarterite infecciosa foi relatada como sendo de 1% ao ano e tem diminuído progressivamente.^2^ A primeira linha de tratamento é a administração de medicamentos antimicrobianos durante várias semanas. Em muitos casos, esta abordagem de gestão produz bons resultados na resolução da vegetação.^3^ No entanto, em alguns casos, a vegetação persiste após o tratamento, e essa vegetação persistente exige uma abordagem de gestão diferente. Habib et al.^4^ recomendaram que o método usual de remoção da vegetação persistente após um regime antibiótico malsucedido seja a cirurgia. A extração de vegetação com aspiradores endovasculares foi relatada em pacientes adultos e adolescentes^5-9^em alguns casos. Além disso, séries de casos e estudos de metanálise^10,11^envolvendo o uso de aspirador de trombos relataram remoção bem-sucedida de uma ou mais massas intracardíacas no ventrículo direito de cardiopatias não congênitas, sem complicações.

Na análise desses estudos, Makdisi et al.^5^ relataram a remoção bem-sucedida de aproximadamente 80% da vegetação aderida à valva tricúspide com aspirador de trombos em paciente adulto jovem que fazia uso de drogas intravenosas.

Por sua vez, Nishii et al.^6^ relataram dois casos de extração percutânea de vegetação. No primeiro caso, a vegetação estava localizada na valva tricúspide e no átrio direito e se desenvolveu em decorrência da colonização dos cabos de desfibriladores implantáveis. O segundo caso foi em paciente portador de marca-passo permanente e a vegetação estava localizada na valva tricúspide e no ventrículo direito. Ambos os casos ocorreram em pacientes idosos que se recusaram a realizar procedimento cirúrgico. Da mesma forma, em um paciente idoso com desfibrilador externo implantável e endocardite, Dahm et al.^7^ realizaram com sucesso a remoção percutânea de duas vegetações.

Koney et al.,^8^ utilizando um aspirador de trombos, removeram com sucesso uma massa intracardíaca no ventrículo direito de um paciente pediátrico com cardiopatia não congênita, sem complicações.

Todos os autores acima demonstraram a viabilidade da remoção da vegetação intracardíaca por acesso percutâneo com dispositivos aspiradores de trombos.

Há um relato de vegetectomia percutânea com alça transcateter em um paciente pediátrico submetido à cirurgia de tetralogia de Fallot.^9^ Da mesma forma, em uma revisão sistemática de 10 relatos envolvendo 88 pacientes submetidos ao sistema de aspiração percutânea assistida por vácuo (AngioVac™), Rusia et al.^10^ relataram que apenas 86 casos (97,7%) foram total ou parcialmente usados com sucesso para redução ou remoção de vegetação antes do eletrodo percutâneo extração. Não houve complicações decorrentes do procedimento de aspiração e não foram relatados casos de mortalidade relacionada ao procedimento.

Em uma metanálise recente,^11^ um total de 49 artigos sobre trombectomia por sucção ou remoção de vegetação usando o sistema AngioVac^TM^ foram publicados. As indicações foram candidatura cirúrgica ruim (81%) ou a necessidade de reduzir o risco de embolia séptica (19%). O fator de risco mais comum foi abuso de drogas intravenosas visto em 45% (20/49) e dispositivos eletrônicos cardiovasculares implantáveis em 45% (20/49). A sobrevivência na alta foi de 93%.

Mais recentemente, foi relatada com sucesso a recuperação percutânea de vegetação em uma bioprótese valvar aórtica em um paciente octogenário com alto risco para cirurgia cardíaca.^12^

Porém, em alguns casos, esse procedimento pode deixar fragmentos residuais, que geralmente remitem com medicamentos antimicrobianos.

Este relato tem como objetivo apresentar o caso de uma menina com endarterite de persistência do canal arterial (PCA) submetida à remoção percutânea de vegetação nesse nível com sucesso e sem complicações, o que ao mesmo tempo criou a oportunidade para o fechamento percutâneo do PCA em um segundo procedimento.

## Relato de Caso

**Figura 1:**
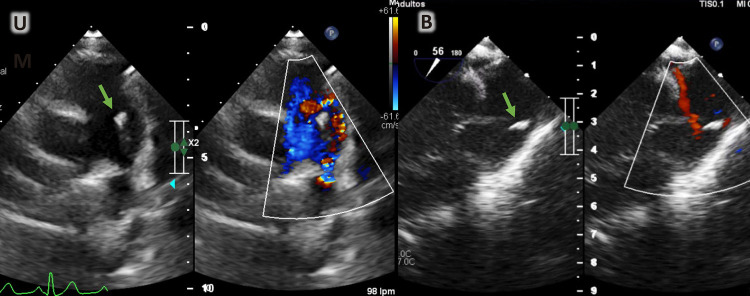
– A) ETT dia 30: podemos observar shunt de PCA (cor vermelha) e vegetação no TP (seta verde). B) ETT. No 40º dia: visualização do fluxo de entrada e saída do ventrículo direito do esôfago médio; Doppler 2D e colorido mostram massa hiperecoica compatível com vegetação (seta verde) no TP.

**Figura 2:**
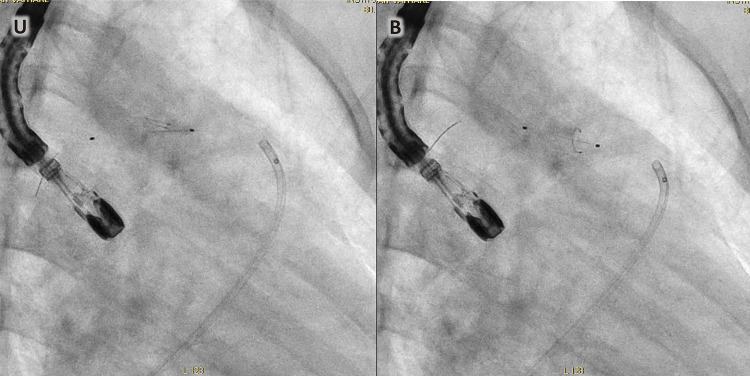
– Medtronic SpiderFx Embolic Protection Device, retração aorta-ducto-tronco pulmonar no 40º dia. A). Dispositivo de recuperação na ampola ductal. B). Dispositivo de recuperação no tronco pulmonar.

Uma menina de oito anos foi internada em nosso Instituto devido a um quadro de febre baixa de 7 meses. Ao exame, foi detectado sopro cardíaco contínuo grau IV/VI. As hemoculturas seriadas mostraram o crescimento de estreptococos nutricionalmente variantes. O ecocardiograma transtorácico (ETT) realizado na admissão revelou a presença de PCA com extremidade pulmonar de 3 mm sem vegetação. Após 30 dias de tratamento antimicrobiano intravenoso com ceftriaxona, o ETT foi repetido, durante o qual a vegetação era claramente apreciada (Figura 1A). No 40º dia, a menina foi submetida a procedimento de retirada de vegetação (Figuras 1B e 2). Inicialmente, a tentativa de recuperação foi realizada com Phenox Thrombectomy Device^TM^ de 2 mm sem sucesso. Posteriormente, um Medtronic SpiderFx Embolic Protection Device^TM^ de 6 Fr. (Figura 2) foi utilizado e a vegetação foi recuperada com sucesso. Primeiro, quatro tentativas de recuperação, fazendo um recuo dos ramos pulmonares até a confluência da artéria pulmonar, falharam. A técnica que permitiu a remoção com sucesso consistiu em fazer um retrocesso da aorta descendente até o tronco pulmonar (TP) através do canal arterial (Figuras 2 e 3). O procedimento foi realizado sem intercorrências em 180 minutos, com perda sanguínea de 50 ml e fluoroscopia de 30,4 minutos. Depois disso, no 58º dia, a criança foi submetida ao fechamento percutâneo da PCA com aparelho Amplatzer^TM^ ADO 8/6 (Figura 4), também sem intercorrências. A menina recebeu alta no 60º dia e a ceftriaxona continuou até então. Após isso, a terapia antimicrobiana continuou com cefixima até o 81º dia. Após o acompanhamento, a criança estava evoluindo sem febre e complicações. Vinte e nove meses depois, os parâmetros cardiovasculares estavam dentro da normalidade e a menina permanecia assintomática. O ETT mostrou uma PCA fechada e nenhum outro achado de importância foi descoberto (Figura 5).

**Figura 3:**
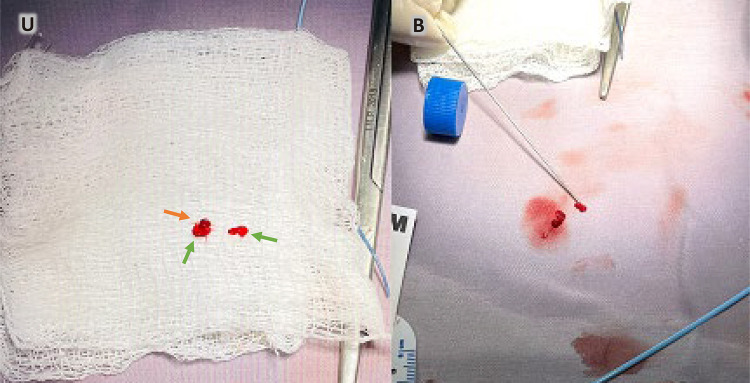
– A e B) Visão macroscópica da vegetação (seta verde) após recuperação e pequeno coágulo (seta laranja).

**Figura 4:**
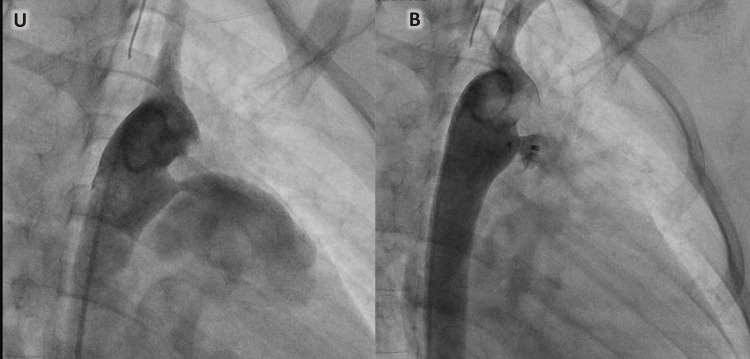
– Angiografia no 58º dia. Podemos ver: A) PCA com extremidade pulmonar de 2,9 mm. B) Dispositivo AmplatzerTM ADO 8/6 fechando PCA.

**Figura 5:**
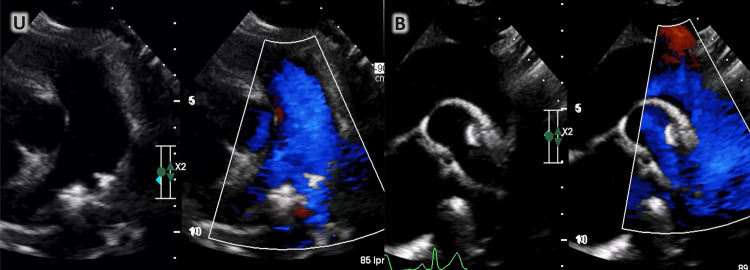
– A) ETT 29 meses depois: podemos observar o fechamento da PCA sem shunt residual com dispositivo AmplatzerTM ADO. A) Vista do eixo curto paraesternal. B). Vista supraesternal.

## Discussão

O presente caso é o primeiro relato de recuperação percutânea bem-sucedida de vegetação crescendo em uma PCA com endarterite em uma paciente pediátrica. A princípio, a retirada da vegetação e o fechamento da PCA foram planejados para serem realizados ao mesmo tempo, porém, devido ao risco de colonização do dispositivo, optou-se por realizar o tratamento percutâneo em dois procedimentos distintos: a extração da vegetação foi realizada inicialmente e em um segundo procedimento, 18 dias após a extração da vegetação, a PCA foi fechada.

A técnica de recuperação foi a seguinte: foi realizada uma angiografia inicial seguida de várias tentativas de recuperação. A primeira tentativa foi realizada com dispositivo de remoção de Stent (Phenox Thrombectomy Device^TM^) sem sucesso. Consequentemente, um filtro carotídeo (Medtronic SpiderFx Embolic Protection Device^TM^) foi utilizado: primeiro, várias tentativas de recuperação fazendo recuo dos ramos pulmonares para a confluência da artéria pulmonar falharam. Optou-se então por cruzar a PCA e realizar um retrocesso pela aorta-ducto-artéria pulmonar sob fluoroscopia e orientação transesofágica, conseguindo a recuperação da vegetação com sucesso após três tentativas (Figura 2).

Nos últimos anos, houve alguns relatos de casos, na literatura, de remoção percutânea de vegetação bem-sucedida e descomplicada no coração direito e esquerdo. A maioria dos casos relatados foi realizada em pacientes adultos e poucos casos foram na população pediátrica.^5-9,12^

O presente caso sugere a utilidade desta técnica para recuperação percutânea de vegetação crescendo em uma PCA em pacientes pediátricos. Acreditamos que este método também pode funcionar na recuperação de vegetação de caule fino. Embora tenha havido alguns relatos de casos de recuperação transcateter de vegetação, essas técnicas só seriam utilizadas com sucesso em casos selecionados. Esta nova técnica, aqui descrita, seria contraindicada em pacientes com lesão tecidual subjacente ou calcificação e não é recomendada em pacientes idosos.

## Conclusão

As diretrizes e/ou recomendações atuais^4,13^no manejo da endocardite/endarterite infecciosa incluem o manejo antimicrobiano e a ressecção cirúrgica em caso de persistência da vegetação. Este caso demonstrou a viabilidade do uso de dispositivo de proteção embólica para recuperação de vegetação em uma PCA com bons resultados e sem complicações. No entanto, mais estudos são necessários para validar esta nova abordagem.
