# Supplementary material for: Recuperação Percutânea de Vegetação em Paciente Pediátrico com Persistência do Canal Arterial: Uma Nova Técnica para Evitar Toracotomia
Source: Arq Bras Cardiol. 2024 Oct 8;121(9):e20140010. [Article in Portuguese] doi: 10.36660/abc.20240010 (PMC11634223; doi:10.36660/abc.20240010)
Supplement: Translation

# Translation: Percutaneous Retrieval of a Vegetation in a Pediatric Patient with Patent Ductus Arteriosus: A New Technique to Avoid Thoracotomy

**DOI:** 10.36660/abc.20240010i

**Authors:** Miguel Fabian Barrerra-Colín, José Luis Colín-Ortiz, Carlos Alfonso Corona-Villalobos

**Affiliations:** 1 Mexico Instituto Nacional de Pediatria, Ciudad De Mexico – Mexico

**Keywords:** Endocarditis, Percutaneous Coronary Intervention, Patent Ductus Arteriosus, Child

## Abstract

Infective endocarditis in the pediatric population is a rare condition that may or may not be associated with a congenital heart disease. Current treatment modalities are based on long-term antibiotic therapy and surgical resection as the first option for cases of persistent vegetation. We present a case of successful percutaneous retrieval of a vegetation in a patent ductus arteriosus, that extended to the pulmonary artery trunk in a pediatric patient. This is the first report in the literature on this type of extraction of vegetation in the ductus arteriosus without thoracotomy.

## Introduction

Intra- or extra-cardiac masses can be tumors, thrombi, or vegetations.^1^ Vegetations can occur in patients without underlying heart disease, but in most cases, they are associated with congenital or acquired heart disease. The incidence of infective endarteritis has been reported to be 1% per year and this has progressively declined.^2^ The first line of treatment is the administration of antimicrobial drugs for several weeks. In many cases, this management approach yields good outcomes achieving the resolution of the vegetation.^3^ However, in some cases, the vegetation persists after the treatment, such persistent vegetation calls for a different management approach. Habib et al.^4^ recommended that the usual method of removing persistent vegetation following an unsuccessful antibiotic regimen is surgery. Vegetation extraction with endovascular aspirators has been reported in adult and adolescent patients^5-9^ in some cases. In addition, case series and metanalysis^10,11^ studies involving the use of a thrombus aspirator have reported successful removal of one or more intracardiac masses in the right ventricle of non-congenital heart disease without complications.

In the analysis of these studies; Makdisi et al.^5^ reported the successful removal of approximately 80% of vegetation adhering to the tricuspid valve with a thrombus aspirator in a young adult patient who had been using intravenous drugs.

On their part, Nishii et al.^6^ reported two cases of percutaneous extraction of vegetation. In the first case, the vegetation was located on the tricuspid valve and right atrium and developed as a result of the colonization of implantable defibrillator leads. The second case was in a patient with a permanent pacemaker and the vegetation was located on the tricuspid valve and right ventricle. Both cases were in elderly patients who refused to undergo a surgical procedure. Similarly, in an older patient with an implantable external defibrillator and endocarditis, Dahm et al.^7^ successfully carried out percutaneous removal of two vegetations.

Koney et al.^8^ using a thrombus aspirator successfully removed an intracardiac mass in the right ventricle of a non-congenital heart disease pediatric patient without complications.

All the authors above demonstrated the feasibility of intracardiac vegetation removal by percutaneous access with thrombus aspirator devices.

There is a report of percutaneous transcatheter snare vegetectomy in a pediatric patient that was undergone tetralogy of Fallot surgery.^9^ Likewise, in a systematic review of 10 reports involving 88 patients who underwent percutaneous vacuum-assisted aspiration system (AngioVac™), Rusia et al.^10^ reported that only 86 cases (97.7%) were completely or partially successfully used to debulk or remove vegetation before percutaneous lead extraction. There were no complications from the aspiration procedure, and no cases of procedure-related mortality were reported.

In a recent metanalysis,^11^ a total of 49 articles on suction thrombectomy or vegetation removal using the AngioVac^TM^ system were published. Indications were poor surgical candidacy (81%) or the need to reduce septic emboli risk (19%). The most common risk factor was intravenous drug abuse seen in 45% (20/49) and cardiovascular implantable electronic devices in 45% (20/49). Survival at discharge was 93%.

More recently, successful percutaneous retrieval of vegetation on an aortic valve bioprosthesis in an octogenarian patient at high risk for cardiac surgery^12^ was reported.

However, in some cases, this procedure can leave residual fragments, which are usually remitted with antimicrobial drugs.

This report aims to present a case of a girl with endarteritis of the patent ductus arteriosus (PDA) who underwent successful percutaneous removal of vegetation at this level without complications, which at the same time, created the opportunity for percutaneous closure of the PDA on a second procedure.

## Case Report

A girl, eight years old, was admitted to our Institute due to a 7-month low-grade fever course. On examination, a grade IV/VI continuous heart murmur was detected. Serial blood cultures depicted the growth of nutritionally variant streptococci. A transthoracic echocardiogram (TTE) performed on admission revealed the presence of a PDA with a pulmonary end of 3 mm without any vegetation. After 30 days of intravenous antimicrobial treatment with ceftriaxone, TTE was repeated, during which vegetation was clearly appreciated (Figure 1A). On the 40^th^ day, the girl was subjected to a vegetation retrieval procedure (Figures 1B and 2). Initially, the retrieval attempt was performed with a Phenox Thrombectomy Device^TM^ of 2 mm without success. Subsequently, a Medtronic SpiderFx Embolic Protection Device^TM^ of 6 Fr. (figure 2) was used and the vegetation was successfully retrieved. First, four retrieval attempts doing a pullback from pulmonary branches to the pulmonary artery confluence failed. The technique that allowed successful removal consisted of doing a pullback from the descending aorta to the pulmonary trunk (PT) through the ductus arteriosus (Figures 2 and 3). The procedure was performed without complications in 180 minutes, with 50 ml volume of blood loss and fluoroscopy of 30.4 minutes. Thereafter, on the 58^th^ day, the child underwent percutaneous closure of PDA with an Amplatzer^TM^ ADO device 8/6 (Figure 4), also without complications. The girl was discharged on the 60^th^day and ceftriaxone continued until this point. After that, antimicrobial therapy continued with cefixime until the 81st day. Upon follow-up, the child was thriving without fever and complications. Twenty-nine months later, cardiovascular parameters were within normal ranges and the girl remained asymptomatic. The TTE showed a closed PDA and no other findings of importance were discovered (Figure 5).

**Figure 1:** – A) TTE on day 30th: we can see a PDA shunt (red color) and vegetation in the PT (green arrow). B) ETE. At day 40th: Mid-esophageal right ventricular Inflow-outflow view; 2D and color Doppler show a hyperechoic mass compatible with vegetation (green arrow) into the PT.

**Figure 2:** – Medtronic SpiderFx Embolic Protection Device, aorta-ductus-pulmonary trunk pullback at day 40th. A) Retrieval device in the ductal ampulla. B) Retrieval device in the pulmonary trunk.

**Figure 3:** – A and B) Macroscopic view of vegetation (green arrow) after retrieval and a little clot (orange arrow).

**Figure 4:** – Angiography on the 58th day. We can see: A) PDA with the pulmonary end of 2.9 mm. B) AmplatzerTM ADO 8/6 device closing PDA.

**Figure 5:** – A) TTE at 29 months later: we can see AmplatzerTM ADO device closure PDA without a residual shunt. A) Parasternal short axis view. B) Suprasternal view.

## Discussion

The present case is the first report on successful percutaneous retrieval of vegetation growing on a PDA with endarteritis in a pediatric patient. At first, the vegetation removal and PDA closure were planned to be performed at the same time, however, due to the risk of device colonization, it was decided to perform the percutaneous treatment in two separate procedures: vegetation extraction was initially performed and in a second procedure, 18 days after vegetation extraction, the PDA was closed.

The retrieval technique was as follows: an initial angiography was performed followed by various retrieval attempts. The first attempt was made using a stent removal device (Phenox Thrombectomy Device^TM^) without success. Consequently, a carotid filter (Medtronic SpiderFx Embolic Protection Device^TM^) was used: first, several retrieval attempts doing pullback from pulmonary branches to the pulmonary artery confluence failed. Then, it was decided to cross the PDA and make a pullback through the aorta-ductus-pulmonary artery under fluoroscopy and transesophageal guidance, achieving the retrieval of the vegetation with success after three attempts (Figure 2).

In recent years, there have been some case reports, in the literature, on successful and uncomplicated percutaneous removal of vegetation in the right and left heart. Most of the cases reported were performed in adult patients and few cases were in pediatric population.^5-9,12^

The present case suggests the usefulness of this technique for percutaneous retrieval of vegetation growing in a PDA in pediatric patients. We think that this method may also work in the retrieval of thin-stemmed vegetation. Even though there have been some case reports on transcatheter retrieval of vegetation, these techniques would only be successfully used in selected cases. This new technique, here described, would be contraindicated in patients with underlying tissue damage or calcification and it is not recommended in older adult patients.

## Conclusion

Current guidelines and/or recommendations^4,13^ in the management of infective endocarditis/endarteritis include antimicrobial management and surgical resection in case of persistence of the vegetation. This case demonstrated the feasibility of using an embolic protection device to retrieve vegetation in a PDA with good results and without complications. However, further studies are needed to validate this new approach.
